# Poster Session I - A63 EUS-GUIDED CELIAC PLEXUS INTERVENTIONS IN PANCREATITIS AND PANCREATIC CANCER: A SYSTEMATIC REVIEW AND META-ANALYSIS OF RANDOMIZED CONTROLLED TRIALS

**DOI:** 10.1093/jcag/gwaf042.063

**Published:** 2026-02-13

**Authors:** M Hu, K Khalaf, Y Yuan, T Nishimura, H Li, M A Bucheeri, G May, J Mosko, N Calo

**Affiliations:** Division of Gastroenterology, St. Michael’s Hospital, University of Toronto, Toronto, ON, Canada; Division of Gastroenterology, St. Michael’s Hospital, University of Toronto, Toronto, ON, Canada; Division of Gastroenterology, St. Michael’s Hospital, University of Toronto, Toronto, ON, Canada; Division of Gastroenterology, St. Michael’s Hospital, University of Toronto, Toronto, ON, Canada; University of Toronto, Toronto, ON, Canada; Division of Gastroenterology, St. Michael’s Hospital, University of Toronto, Toronto, ON, Canada; Division of Gastroenterology, St. Michael’s Hospital, University of Toronto, Toronto, ON, Canada; Division of Gastroenterology, St. Michael’s Hospital, University of Toronto, Toronto, ON, Canada; Division of Gastroenterology, St. Michael’s Hospital, University of Toronto, Toronto, ON, Canada

## Abstract

**Background:**

Chronic abdominal pain is a major source of morbidity for patients with chronic pancreatitis and pancreatic cancer, and effective pain management options are limited. Endoscopic ultrasound (EUS)-guided celiac plexus interventions are minimally invasive procedures designed to alleviate severe abdominal pain associated with these conditions, however, data reporting the effect of these interventions are scarce and based in small reports.

**Aims:**

This systematic review and meta-analysis evaluates the effectiveness and safety of EUS-guided celiac plexus interventions for managing abdominal pain in patients with chronic pancreatitis and pancreatic cancer.

**Methods:**

A comprehensive search of OVID MEDLINE, EMBASE, and Cochrane CENTRAL was conducted from inception to September 1^st^, 2025. We included full-text randomized controlled trials (RCTs) evaluating EUS-guided celiac plexus block (CPB), celiac plexus neurolysis (CPN), or celiac ganglion neurolysis (CGN) in patients with chronic pancreatitis or pancreatic cancer. The primary outcome was the proportion of patients experiencing pain relief. Secondary outcomes included duration of pain relief, reduction in opioid consumption, and adverse events.

**Results:**

14 RCTs (n = 596 patients) met the inclusion criteria. 4 studies evaluated EUS-CPB in patients with chronic pancreatitis, and 10 studies evaluated EUS-CPN or CGN in patients with pancreatic cancer. The overall pooled proportion of patients with pain relief was 70% (95% CI: 0.60-0.79), with significant heterogeneity (I^2^=80%). In subgroup analyses, the pooled proportion of pain relief was 73% (95% CI: 0.50-0.91) for patients with chronic pancreatitis and 68% (95% CI: 0.57-0.78) for patients with pancreatic cancer. The mean duration of pain relief, reported in 4 studies, was 89.8 days, and 7 studies reported on changes in opioid consumption. Serious adverse events were infrequent; 3 studies reported a total of 3 major events (1 upper gastrointestinal bleed, 1 irreversible paralysis, and 1 hematoma). Furthermore, 2 studies reported that 6 patients required hospitalization or an emergency department visit for severe abdominal pain.

**Conclusions:**

EUS-guided celiac plexus interventions provide significant pain relief for patients with abdominal pain secondary to chronic pancreatitis and pancreatic cancer. These findings support their use as a key component in a multimodal pain management strategy. However, significant heterogeneity across studies and small sample sizes highlights the need for larger, well-designed RCTs. Future research should focus on standardizing techniques, identifying predictors of response, and evaluating long-term effects on opioid consumption and quality of life to better define the role of these interventions.

**Funding Agencies:**

None

